# Early brain injury linearly correlates with reduction in cerebral perfusion pressure during the hyperacute phase of subarachnoid hemorrhage

**DOI:** 10.1186/s40635-014-0030-1

**Published:** 2014-11-30

**Authors:** Serge Marbacher, Volker Neuschmelting, Lukas Andereggen, Hans Rudolf Widmer, Michael von Gunten, Jukka Takala, Stephan M Jakob, Javier Fandino

**Affiliations:** Cerebrovascular Research Laboratory of the Department of Intensive Care Medicine, University Hospital and University of Bern, 3010 Bern, Switzerland; Department of Neurosurgery, Kantonsspital Aarau, 5000 Aarau, Switzerland; Department of Neurosurgery, University Hospital Cologne, 50937 Cologne, Germany; Department of Neurosurgery, Bern University Hospital, University of Bern, 3010 Bern, Switzerland; Institute of Pathology Länggasse, 3012 Bern, Switzerland

**Keywords:** Cerebral perfusion pressure, Intracranial pressure, Early brain injury, Rabbit, Subarachnoid hemorrhage, Neurodegeneration

## Abstract

**Background:**

It is unclear how complex pathophysiological mechanisms that result in early brain injury (EBI) after subarachnoid hemorrhage (SAH) are triggered. We investigate how peak intracranial pressure (ICP), amount of subarachnoid blood, and hyperacute depletion of cerebral perfusion pressure (CPP) correlate to the onset of EBI following experimental SAH.

**Methods:**

An entire spectrum of various degrees of SAH severities measured as peak ICP was generated and controlled using the blood shunt SAH model in rabbits. Standard cardiovascular monitoring, ICP, CPP, and bilateral regional cerebral blood flow (rCBF) were continuously measured. Cells with DNA damage and neurodegeneration were detected using terminal deoxynucleotidyl transferase dUTP nick end labeling (TUNEL) and Fluoro-jade B (FJB).

**Results:**

rCBF was significantly correlated to reduction in CPP during the initial 15 min after SAH in a linear regression pattern (*r*^2^ = 0.68, *p* < 0.001). FJB- and TUNEL-labeled cells were linearly correlated to reduction in CPP during the first 3 min of hemorrhage in the hippocampal regions (FJB: *r*^2^ = 0.50, *p* < 0.01; TUNEL: *r*^2^ = 0.35, *p* < 0.05), as well as in the basal cortex (TUNEL: *r*^2^ = 0.58, *p* < 0.01). EBI occurred in animals with severe (relative CPP depletion >0.4) and moderate (relative CPP depletion >0.25 but <0.4) SAH. Neuronal cell death was equally detected in vulnerable and more resistant brain regions.

**Conclusions:**

The degree of EBI in terms of neuronal cell degeneration in both the hippocampal regions and the basal cortex linearly correlates with reduced CPP during hyperacute SAH. Temporary CPP reduction, however, is not solely responsible for EBI but potentially triggers processes that eventually result in early brain damage.

**Electronic supplementary material:**

The online version of this article (doi:10.1186/s40635-014-0030-1) contains supplementary material, which is available to authorized users.

## Background

Acute intracranial pathophysiological events occurring at the time of acute subarachnoid hemorrhage (SAH) and their sequelae during the first days thereafter have recently received increased attention. Clinical and experimental work has long demonstrated that early brain injury (EBI) after SAH plays an important role in the disease pathophysiology [1-3]. A large clinical trial showed that reduction of delayed cerebral vasospasm failed to improve clinical outcomes [4]. Hence, interventions acting early in the disease course continued to gain in importance [5-7]. Despite increased research efforts in recent years, there is still relatively little known about what triggers pathophysiological mechanisms that result in EBI after SAH [8,9].

EBI is an umbrella term that embraces consequences of complex pathophysiological mechanisms that occur as a result of the initial bleed [8] and are unlikely to be solely responsible for early ischemic damage. It is evident that co-factors such as microvascular filling defects [10], breakdown of ionic homeostasis [11], blood brain barrier disruption, microarterial narrowing [12], and decreased bilateral regional cerebral blood flow (rCBF) worsen ischemia. To date, there has been little agreement on what triggers these processes which ultimately result in EBI after SAH.

It has been demonstrated that rapid and large increase in intracranial pressure (ICP) leads to more severe acute pathophysiologic (greater rCBF reduction) and histological changes (increased in Fluoro-jade B (FJB)- and terminal deoxynucleotidyl transferase dUTP nick end labeling (TUNEL)-positive cells) after experimental SAH [13]. However, in this experimental setting, the extent of SAH was macroscopically more pronounced (reflected in a nearly four times higher hemoglobin concentration in the subarachnoid space basal brain areas) in animals with larger increase in ICP. And since subarachnoid blood *per se* is well known to cause direct brain damage, late rCBF reduction, and neuronal and astrocytic apoptosis independent of initial ICP increase [14-18], it still remains a matter of debate whether ICP increase or the extent of subarachnoid blood represents one of the main causes for increased EBI after SAH.

In order to investigate how peak ICP, extent of subarachnoid blood, and hyperacute depletion of cerebral perfusion pressure (CPP) may correlate with the onset of EBI, we used a blood shunt SAH model to control and simulate various degrees of ICP increase.

## Methods

A total of 21 3-month-old female New Zealand White rabbits weighing 2.4 to 4.3 kg were used in this study. The study was incorporated as a subproject of ongoing experimental studies and performed in accordance with the National Institutes of Health guidelines for the care and use of experimental animals and with the approval of the Animal Care Committee of the Canton of Bern, Switzerland (approval #107/09) [19]. The animals were housed in groups (two to four animals per cage) at 22°C to 24°C under a 12-h light-dark cycle with free access to food and tap water.

### Study design

Sixteen rabbits underwent various degrees of ICP-controlled (range 40 to 120 mmHg) SAH to generate a spectrum of ICP values as described in more detail below. Five animals served as sham-operated controls. All surgical procedures were performed under sterile conditions at the Experimental Surgical Institute, Department of Clinical Research, Bern University Hospital, Bern, Switzerland. A veterinary anesthesiologist monitored the animals during surgery and throughout anesthetic recovery.

### Anesthesia, clinical observation, and sacrifice

Induction of general anesthesia was performed by subcutaneous administration of ketamine (30 mg/kg; Ketalar, 50 mg/ml, Pfizer AG, Zurich, Switzerland) and xylazine (6 mg/kg; Xylapan, 20 mg/ml, Vetoquinol AG, Bern, Switzerland) and continued intravenously. Room air-enriched oxygen was provided to the spontaneously breathing animals. The animals underwent clinical observation during anesthetic recovery (first 3 h) and from then on every 6 h. Neurological status was graded at 3, 6, 12, 18, and 24 h post-SAH according to a four-point grading system [20]: grade 1, no neurological deficit; grade 2, minimal or suspected neurological deficit; grade 3, mild neurological deficit without abnormal movement; and grade 4, severe neurological deficit with abnormal movement. Euthanasia was performed 24 h post-SAH induction under the same anesthesia as previously described, by intra-arterial bolus injection of sodium thiopental (40 mg/kg) (Pentothal®, Ospedalia AG, Hünenberg, Switzerland).

### SAH induction, instruments, and data acquisition

Since our primary research question required an entire spectrum of various degrees of SAH, measured as ICP increase, we were dependent on tight control of ICP. Hence, we ultimately have chosen the blood shunt model which has been validated for that purpose in various species [21-24]. The model and techniques were used to induce SAH in rabbits as described previously [19]. Briefly, on day 0, the cisterna magna was punctured with a pediatric spinal access needle (22 G × 40 mm) and connected via a pressure tube and interposed three-way stopcock to the subclavian artery. The three-way stopcock was used for blood pressure measurement, as a blood sampling port, and to allow regulation of the bleeding. Neuromonitoring including an ICP monitor catheter tip (OLM Intracranial Pressure Monitoring Kit, Camino, Model 110-4B, Camino Laboratories, San Diego, CA, USA) and two laser-Doppler flowmetry fine needle probes (MNP110XP, 0.48-mm diameter, Oxford Optronix Ltd., Oxford, UK) which were positioned in the olfactory bulb and bilateral frontal lobe according to outer skull landmarks was done [25].

Standard cardiovascular monitoring (mean arterial blood pressure (MABP), heart rate, electrocardiogram, end-tidal CO_2_, and SaO_2_) was performed at a sampling rate of 100 Hz (Datex S5 Monitor, GE Medical Systems CH, Glattbrugg, Switzerland), and the data were transferred via the analog output interface to an analog-digital converter/data logger, stored (Biopac MP100 and acqKnowledge version 3.8.1; BIOPAC Systems, Inc., Goleta, CA, USA), and processed for pre-analysis using scripting software matlab (Mathworks 130 Inc., Natick, MA, USA). Pressures were zeroed to the level of the heart before and after each session, and pressure calibration of the AD converter and data-logging system was done once before the series started. Arterial blood gas status was analyzed (ABL 725, Radiometer, Copenhagen, Denmark) before SAH induction.

Baseline values were measured during a time period of 6 min. SAH was initiated by opening the blood shunt to let blood stream into the atlanto-occipital cistern under arterial pressure. MABP and bilateral rCBF were recorded for 15 min after initiation of SAH. Closure of the stopcock interposed in the shunt allowed creation of various degrees of ICP increase (40 to 120 mmHg) and subsequent CPP depletion. Most of the animals were exposed sequentially to ‘Spontaneous SAH’ (*n* = 10). After opening of the shunt, ICP increases without any intervention until reaching a plateau. If this plateau phase was maintained for more than 10 s, the shunt was closed. The shunt was also closed if ICP decrease occurred spontaneously (no later than 30 s from start of the plateau phase; we therefore did not allow for potential rebleeding). Most of the animals with spontaneous SAH thus suffered severe SAH with high ICP values. In a minority of cases, spontaneous plateaus were reached at rather low ICP levels. These mild cases of SAH probably occurred due to premature thrombosis within the shunt system.

**Figure 1:**
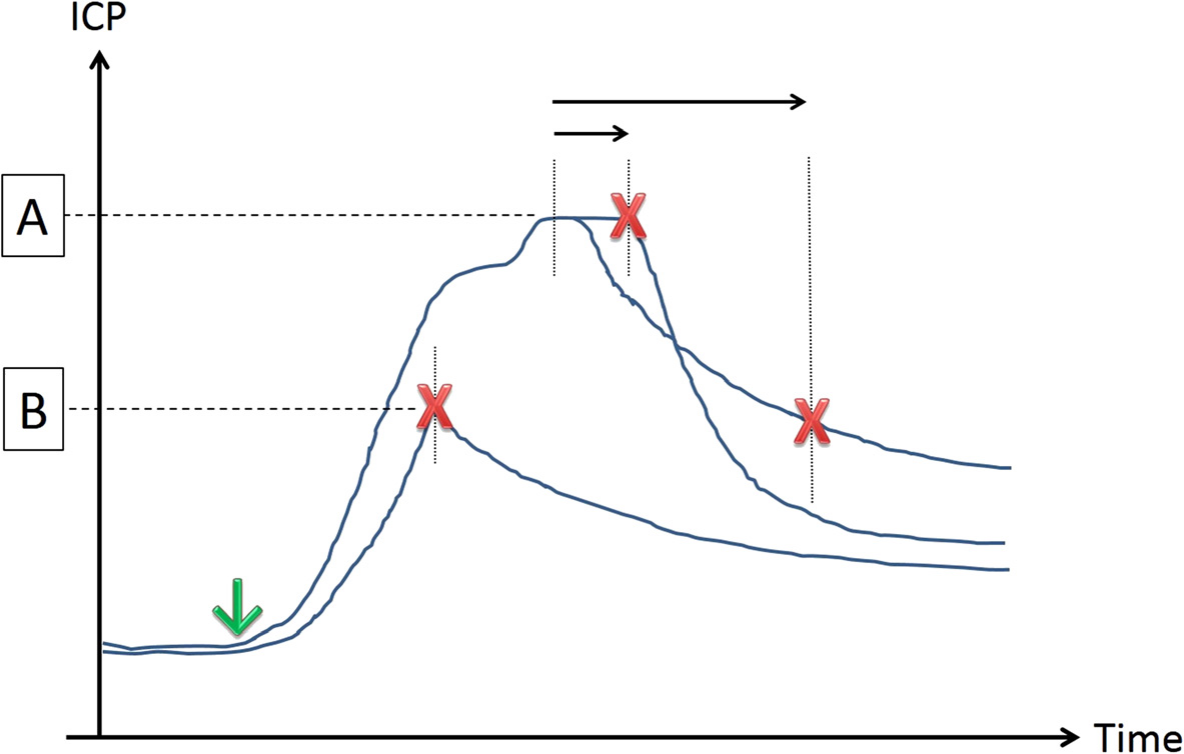
**Spontaneous and ICP controlled SAH.** Scheme depicts start (down arrow) and stop (red X) procedures of shunt-induced SAH. **(A)** ‘Spontaneous SAH’. The shunt is closed after ICP either reached a plateau (10 s; short horizontal arrow) or started to decrease (30 s; long horizontal arrow). **(B)** ‘Controlled SAH’. The shunt is closed at any level prior to development of ICP plateau.

However, to test our hypothesis, we needed the entire spectrum of different ICP values. Therefore, we performed ‘Controlled SAH’ to add missing SAH severities by means of ICP increase (*n* = 6). These animals were randomized to planed maximal ICP values. Target peak values were achieved by closure of the shunt during ICP increase within the range of minimal (40 mmHg) and most severe (120 mmHg) spontaneous ICP increase. An overview of SAH start and various stop procedures of shunt-induced SAH are given in Figure 1.

### Tissue processing, histology, and histochemistry

Under general anesthesia, intracardiac perfusion-fixation was carried out on day 1 after SAH at room temperature with 400 ml of 0.1 M phosphate-buffered solution (PBS) followed by 400 ml fixative (4% paraformaldehyde in 0.1 M PBS, pH 7.3). The brains were removed from the skull, and the basal and hemispheric surfaces were analyzed to identify accumulated blood clots and distribution of subarachnoid blood. The severity of SAH was categorized as reported previously with slight modifications [26] as follows: 0: no blood; 1: minimal blood; 2: moderate blood clot (basal arteries visible); and 3: massive blood clot (visual obliteration of basal arteries). The summed score of each cistern (range: 0 to 12) determined the final grade of controls (0), mild (1 to 4), moderate (5 to 8), or severe (9 to 12) SAH (Additional file 1: Figure S1).

The brains were then immersed in the fixative overnight and cryoprotected at 4°C followed by immersion in 15% sucrose in 0.1 M PBS. The brains were cut into four blocks between the forebrain (olfactory bulb) and cerebellum, embedded in paraffin, and cut into consecutive 7-μm sections. The cut surface of block one was placed through the cortical punch defect of the ICP and rCBF probes. The first section of blocks two to four was stained with hematoxylin and eosin, and the most representative fields containing the hippocampus and basal cortex (BC) were selected for additional cuts of nine consecutive sections used for histochemical analysis.

Cells with damaged deoxyribonucleic acid (DNA) and neurodegeneration secondary to ischemia were detected using the TUNEL method (Roche Diagnostics AG, Rotkreuz, Switzerland) and FJB (Millipore AG, Zug, Switzerland). Nuclei were counterstained with 4',6-diamidino-2-phenylindole (DAPI) (Roche Diagnostics AG, Basel, Switzerland). An observer (L.A.) blind to sample identity counted the number of co-localized cells in regions of interest defined on coronal sections for each hemisphere in the basal cortex (0.9 mm^2^) and along the hippocampal cornu ammonis regions CA1 and CA3 (each 0.9 mm).

### Data analysis and statistical methods

Statistical analysis was performed using IBM SPSS statistical software version 20.0 (IBM Corp., New York, NY, USA) and processed for pre-analysis using Matlab scripting software (Mathworks Inc., Natick, MA, USA). Areas under the curve (AUC) were calculated based on the trapezoidal rule on the 100-Hz acquisition data set before any further post-processing. ANOVA regression analysis was used for calculation of correlations between effects of SAH on hemodynamics, rCBF, ICP, CPP, number of FJB- and TUNEL-positive cells, and the amount of subarachnoid blood (SAH blood score), respectively. The time buckets chosen for analyses (1.5, 3, and 6 min after SAH) were predetermined (*a priori*) based on the pathophysiology of the rabbit shunt model (peak ICP increase within 1 to 2 min, maximal CPP depletion within the first 3 min, ICP steady-state values within 5 to 10 min) [19]. Based on the relative CPP depletion during the first 3 min of SAH, the animals were assigned *post hoc* to one of three groups of hemorrhage severity: mild (*n* = 5; relative CPP < 0.25), moderate (*n* = 3; relative CPP > 0.25 but < 0.4), and severe (*n* = 4; relative CPP > 0.4). The groups' mean cell counts of FJB- and TUNEL-positive cells of both hemispheres were then compared among those and to the control group using one-way ANOVA and Bonferroni *post hoc* testing. Values are expressed as means of each group ± SD. Data from neurological deficit scores and subarachnoid blood scores are given as median and range. A probability value of <0.05 was considered statistically significant. The strength of linear correlations between the variables was expressed by the linear regression coefficient (*r*) and its squared value *r*^2^.

## Results

### Mortality, morbidity, and neurological status

The mortality of SAH animals was 25% (4 out of 16 rabbits). All of the animals that died were allocated to spontaneous SAH and died shortly after initiation of the bleeding due to respiratory arrest and severe bradycardia. Surviving animals (total *n* = 12; n = 6 ‘Spontaneous SAH’ and *n* = 6 ‘Controlled SAH’) demonstrated a uniform early post-SAH clinical course, with slow recovery within the first 3 h (median neurological score: 3; range 2 to 4). Most SAH animals (*n* = 7) recovered completely within 24 h post-surgery (median neurological score: 1; range 1 to 4). Control animals showed uneventful recovery within 3 to 6 h.

### Interplay between MABP, ICP, CPP, and rCBF

**Figure 2:**
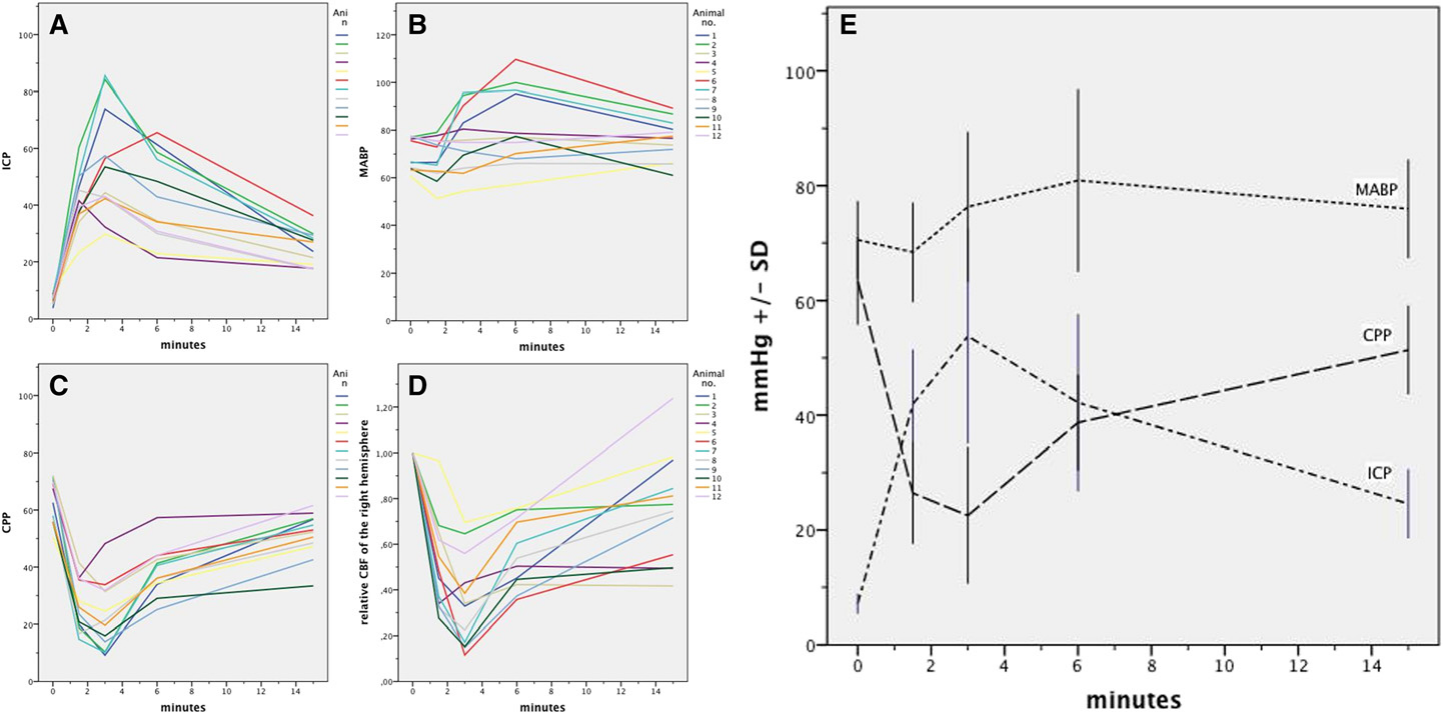
**ICP, MABP, CPP and rCBF time-course and values of all SAH animals.** All SAH animals demonstrated a rapid increase in ICP **(A)**. Marked increase of MABP **(B)** occurred during the peak phase of CPP **(C)** and rCBF **(D)** depletion. Mean values ±SD are presented in **(E)**. ICP, MABP, and CPP values are displayed in mmHg. rCBF of the right hemisphere is given as mean relative baseline values within 15 min.

All SAH animals demonstrated a rapid increase in ICP with a corresponding marked decrease in CPP and rCBF. The increase of MABP during the peak phase of ICP (Cushing reflex) was more pronounced than the further increase in ICP, resulting in rapid recovery of CPP to 81.5% ± 12% of baseline values within 15 min. The mean relative rCBF depletion of both hemispheres was significantly correlated to reduction in CPP during the initial 15 min after SAH in a linear regression pattern (reg coeff *r* = 0.82, *r*^2^ = 0.68, *p* < 0.001). Time course and individual values of ICP, MABP, CPP, and local rCBF are given in Figure 2.

### Gross examination of brain and histology

**Figure 3:**
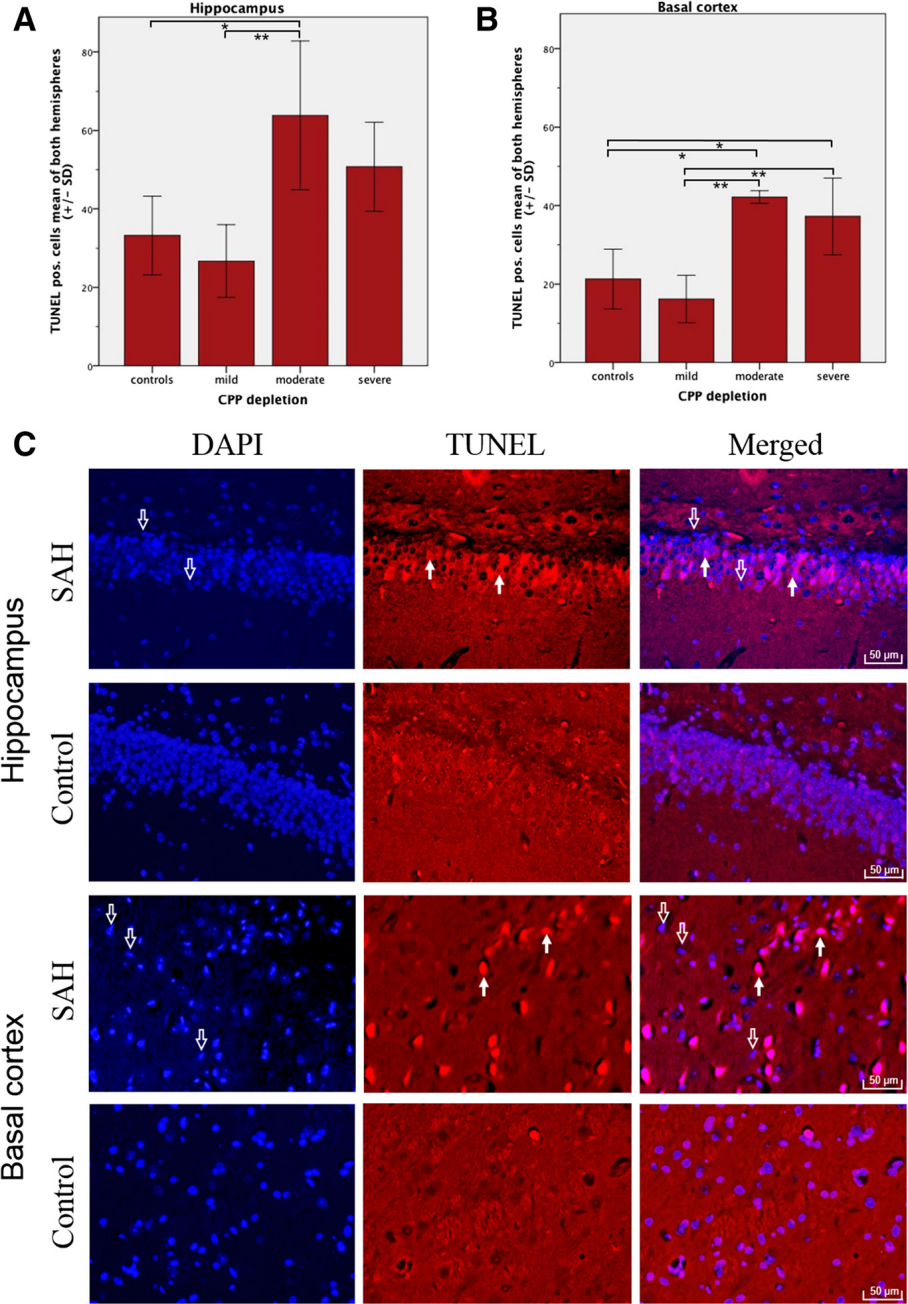
**Cells with DNA damage in hippocampus and basal cortex.** Bar graphs demonstrate mean TUNEL-positive cell count of both hemispheres (±SD) subdivided into controls (*n* = 5) and different degrees of CPP depletion (mild, *n* = 5; moderate, *n* = 3; severe, *n* = 4). Quantification was performed by counting TUNEL-positive cells co-localizing with DAPI (blue) in regions of interest in the hippocampus **(A)** and basal cortex **(B)**. Histochemistry **(C)**: Co-localization with DAPI (left column) confirmed that TUNEL-positive staining (middle column) was generally in the nucleus (right column). Hollow arrows show DAPI-positive nuclear staining (blue). Filled arrows identify TUNEL-positive cells. Scale bars = 50 μm. *p < 0.05. **p < 0.01.

**Figure 4:**
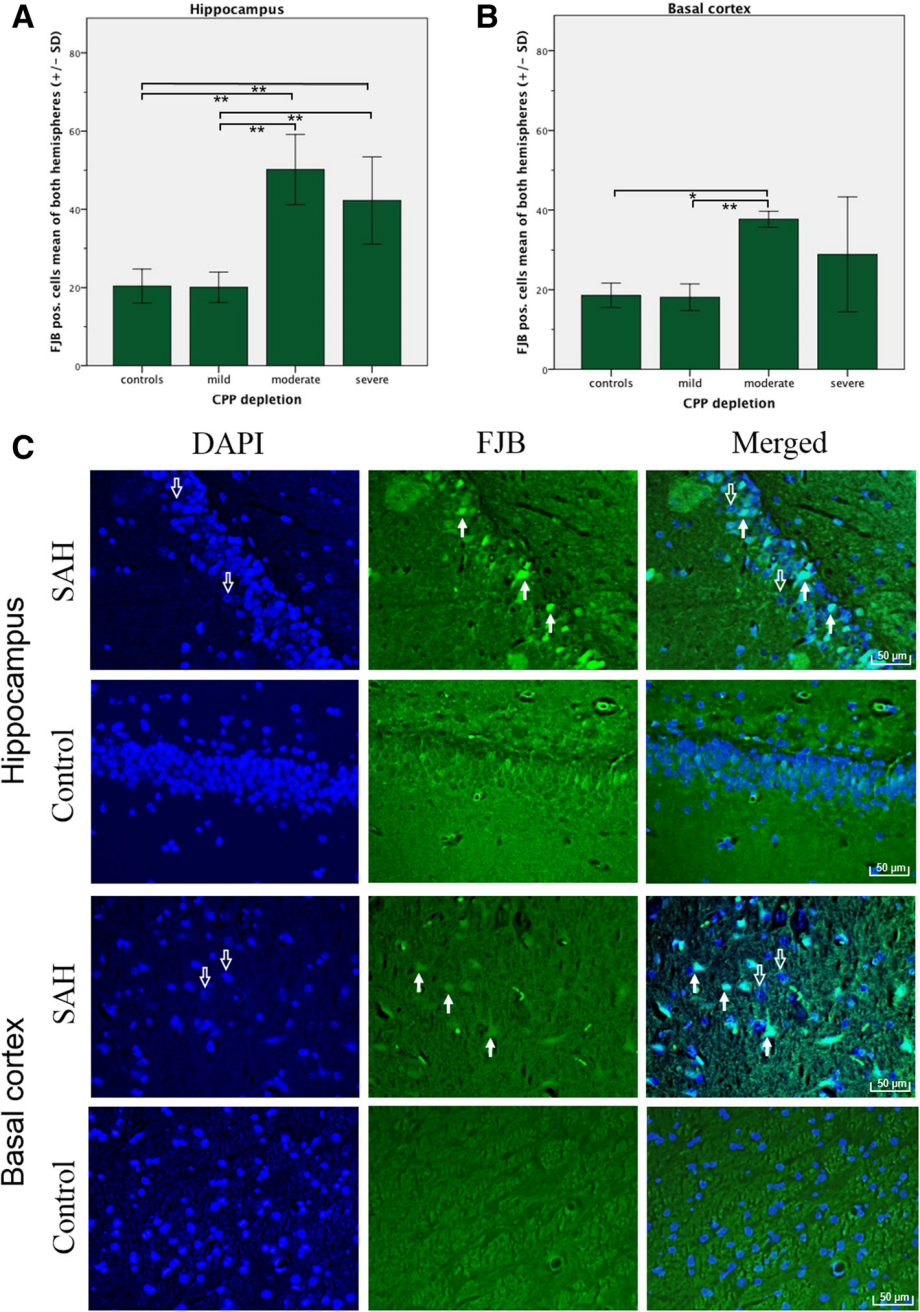
**Neurodegeneration in hippocampus and basal cortex** ***.*** Bar graphs demonstrate mean FJB-positive cell count of both hemispheres (±SD) subdivided into controls (*n* = 5) and different degrees of CPP depletion (mild, *n* = 5; moderate, *n* = 3; severe, *n* = 4). Quantification was performed by counting FJB-positive cells co-localizing with DAPI (blue) in regions of interest in the hippocampus **(A)** and basal cortex **(B)**. Histochemistry **(C)**: Co-localization with DAPI (left column) confirmed that FJB-positive staining (middle column) was generally in the nucleus (right column). Hollow arrows show DAPI-positive nuclear staining (blue). Filled arrows identify FJB-positive cells. Scale bars = 50 μm. *p < 0.05, **p < 0.01.

There were no complications related to wound healing, cerebrospinal fluid leakage, or infections along the frontal osteotomy sites, subclavian skin incision, or nuchal cisternal injection point. Twenty-four hours post-SAH, all surviving rabbits (*n* = 12) demonstrated extensive coagulated diffuse subarachnoid blood in the chiasmatic and pre-chiasmatic cisterns (I: 2; 1 to 3), basal cistern (II: 3; 2 to 3), prepontine and interpeduncular cisterns (III: 3; 1 to 3), and cistern magna (IV: 2; 1 to 3), resulting in moderate (*n* = 2, 7; 7), and severe (*n* = 10, 10 to 12) grades of SAH. No subarachnoid blood was observed in control animals (*n* = 5). Cells with DNA damage and neurodegeneration by means of TUNEL- and FJB-positive cells were detected in the basal cortex regions and the hippocampus (CA1 and CA3) of both hemispheres in all animals. Merged co-localization with DAPI confirmed that TUNEL- and FJB-positive staining was generally located in the nucleus (Figures 3 and 4). Animals exposed to mild CPP depletion showed no differences in the number of cells with DNA damage and neurodegeneration irrespective of location when compared with the control animals (Figures 3 and 4). However, those animals exposed to moderate CPP depletion demonstrated significantly more TUNEL- and FJB-positive stained cells in the hippocampus formation as well as in the BC region than the mild SAH group or the control animals. The mean cell counts of the animals that suffered severe SAH did not statistically differ from those animals exposed to moderate CPP depletion, irrespective of location (Additional file 2: Tables S1 and S2).

### Correlation between CCP depletion, SAH blood score, peak ICP, and EBI

**Figure 5:**
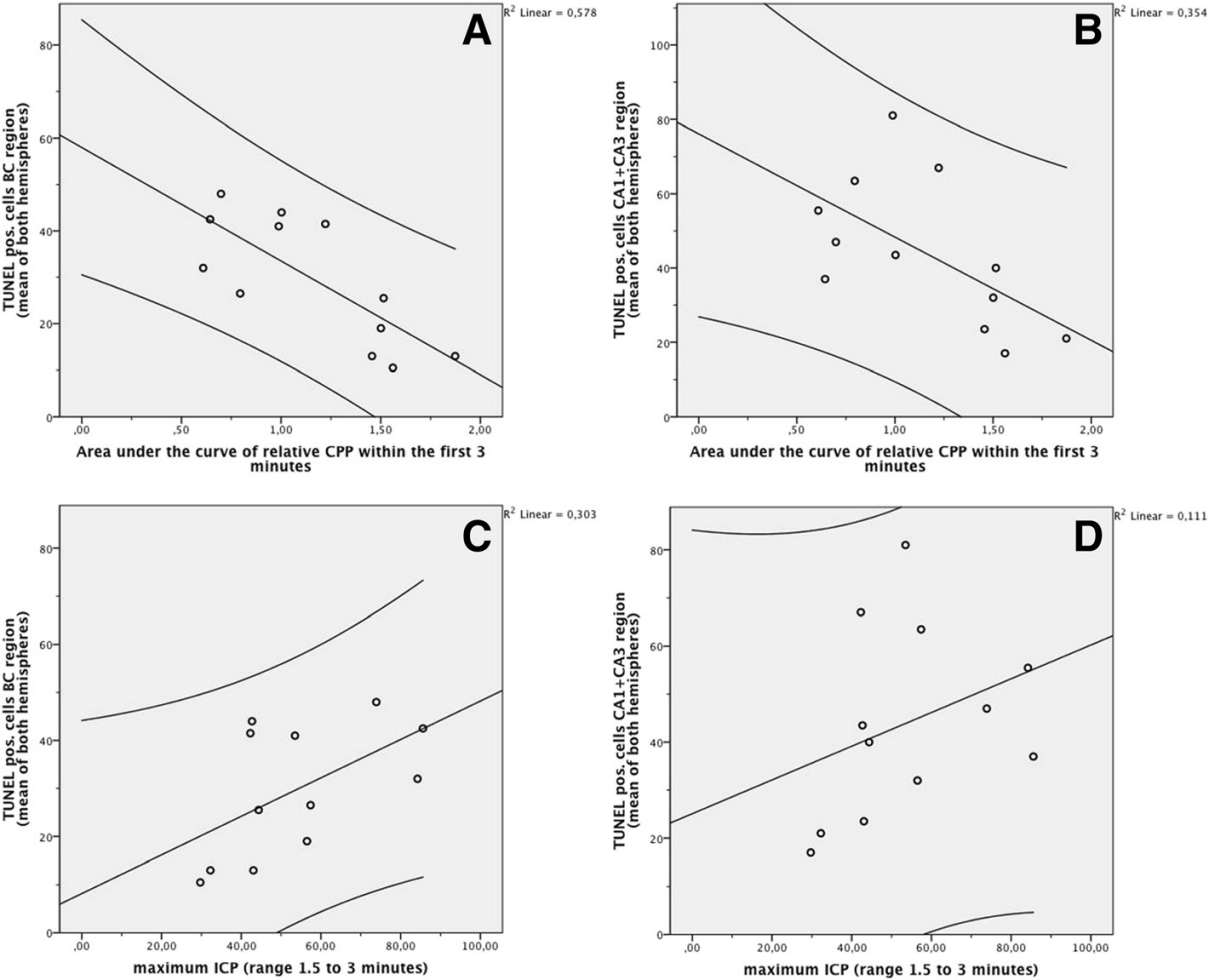
**Correlation between hyperacute CPP reduction/peak ICP and early brain injury** ***.*** A significant positive linear correlation between CPP reduction within the first 3 min after SAH and the total number of the mean of left and right hemispheres TUNEL-positive cells was found in both the basal cortex region **(A)** as well as the hippocampus formation **(B)**, revealing that neuronal cell death and neurodegeneration are linked to the severity of temporary CPP shortage during the first 3 min of SAH. Peak ICP did not significantly correlate with the number of cells with DNA neither in the basal cortex **(C)** nor in the hippocampal **(D)** regions. Graphs include the 95% confidence intervals.

A significant positive linear correlation between CPP reduction within the first 3 min after SAH and the total number of TUNEL- and FJB-positive cells (means of left and right hemispheres) was found in CA1 and CA3 regions (*r*^2^ = 0.51, *p* < 0.01 for the FJB-positive cells and *r*^2^ = 0.35, *p* < 0.05 for the TUNEL-positive cells, respectively) as well as in the basal cortex region for the TUNEL-positive cells (*r*^2^ = 0.58, *p* < 0.01). There was no linear correlation, however, between the relative CPP's area under the curve within the first 3 min and FJB-positively stained cells in the basal cortex region (*r*^2^ = 0.24, *p* > 0.1). The more severe the temporary CPP reduction, the more pronounced seemed the neuronal cell death and neurodegeneration (Figure 5).

There were no linear correlations between the SAH blood score and the relative CPP depletion within the first 3 min and between the SAH blood score and relative CPP at the time of maximal depletion (Additional file 1: Figure S2). There was also no linear correlation between SAH blood score and the total amount of TUNEL- or FJB-positive cells (means of both hemispheres). These findings hold true for both the hippocampus formation and basal cortex region (Additional file 1: Figure S3). Peak ICP did not significantly correlate with any of our neuronal cell damage parameters (*p* > 0.05).

## Discussion

The results of this study demonstrate that the more severe the CPP shortage during the hyperacute phase of SAH, the more pronounced was the number of cells with DNA damage and the degree of neurodegeneration in the hippocampal region and the number of cells with DNA damage in the basal cortex 24 h after experimental SAH. The findings of early neuronal damage were independent of peak ICP and the amount of subarachnoid blood. Using the ICP-controlled shunt model, the results extend prior findings [13-18] and suggest that CPP depletion at the time of SAH potentially triggers processes that eventually result in EBI after SAH.

Although significant, the correlation between hippocampal TUNEL- and FJB-positive cells (means of left and right hemispheres) and hyperacute CPP depletion was weak (*r*^2^ = 0.35 and *r*^2^ = 0.51). In the basal cortex region, the correlation between CPP shortage within the first 3 min and TUNEL-positive cells was slightly stronger (*r*^2^ = 0.58) but did not reach statistical significance for FJB-positive cells. One explanation for the absent correlation of FJB-positive cells could be a mismatch of TUNEL and FJB staining of neurons that underwent oxidative stress [13,27]. Another explanation can be the lack of specificity of FJB [28]. Additional detection of apoptosis by measurement of apoptosis-related proteins would have improved the quantification of cell death, and double labeling of FJB with NeuN would have increased the specificity for detection of neuronal degeneration.

Due to animal welfare regulations, the study was performed with adult female rabbits only. Using female animals carries the risk that estrogens may attenuate SAH-induced apoptosis [29]. The amount of neuronal cell death may also depend on gender and chosen injection anesthesia. It remains unknown whether and to what extent the use of ketamine compromised pathophysiological parameters and neuronal cell death after SAH. Nevertheless, the impact of moderate CPP compromise on cells with DNA damage is highlighted by the differences observed between SAH animals and controls.

### Contribution of acute global cerebral ischemia to EBI after SAH

Clinical observations have long emphasized the important relationship between increase in ICP within the first minutes after aneurysm rupture and occurrence of cerebral ischemia [30]. Although we found a positive correlation between CPP reduction and EBI, it is unlikely that the temporary perfusion shortage is solely responsible for the early neuronal damage detected.

The present data demonstrate that neurodegeneration and neuronal cell death occurred not only in animals with severe acute global cerebral ischemia but also in subjects with moderate CPP reduction. Furthermore, neuronal cell death was equally detected in regions that are less susceptible to ischemia (basal cortex) and regions most vulnerable to ischemic stress (hippocampus). Thus, it can be hypothesized that in addition to temporary CPP reduction during hyperacute SAH, other mechanisms - probably triggered by initial ICP increase and subsequent CPP depletion - are likely responsible for focal and global perfusion deficits and subsequent ischemic damage in the early phase after SAH.

Processes such as acute vasoconstriction (large and small parenchymal vessels) [12,31], perivascular swelling (intra- and extracellular edema) [32], microvascular filling defects (detachment of endothelial cells, platelet aggregation, and microthrombosis) [33], breakdown of ionic homeostasis (increase in extracellular glutamate and cortical spreading depression) [11], and decreased cerebral blood flow (CBF) (probably as secondary response to decreased cerebral metabolic rate and decreased spontaneous electrical activity) are all likely to worsen ischemia. It remains largely unexplored to what extent these mechanisms contribute to EBI and how they are connected among each other.

There were no differences in the number of cells with DNA damage and neurodegeneration between animals that suffered mild CPP challenge (relative CPP < 0.25) and control animals. Although the number of animals in the *post hoc* stratified groups is small, the analysis suggests that the threshold for triggering early brain injury lies between 25% and 40% of CPP depletion during the hyperacute phase of SAH.

### Main triggers of mechanisms that result in EBI after SAH

To date, there has been little discussion about the triggering event for the mechanisms eventually leading to EBI after SAH. The mechanisms that are made responsible for EBI are believed to be activated as early as the aneurysm rupture. Blood streams under arterial pressure into a closed cranium causing rapid rise in ICP and marked CPP depletion which in turn reduces CBF. Experimental clarification of whether an initial global increase in ICP (respectively CPP reduction) or extravasated blood triggers the early pathophysiological sequelae causing EBI was inherently complicated by the lack of models that allow precise control of ICP, or CPP reduction, during acute SAH.

In a comparison of two different SAH rat models, a rapid and large increase in ICP led to more severe acute pathophysiologic (decrease in rCBF) and histological changes (increased in FJB- and TUNEL-positive cells) than minor changes in ICP [13]. However, macroscopic examination also demonstrated significantly higher amounts of subarachnoid hemoglobin in the group with the greater ICP increase and therefore did not confirm whether ICP increase or the amount of subarachnoid blood represents the main cause of acute SAH sequelae.

It has been demonstrated that subarachnoid blood can cause direct brain damage, late rCBF reduction, and neuronal and astrocytic apoptosis independent of initial ICP increase [14-18]. However, we could not establish any relationship between the amount of subarachnoid blood and the degree of early (24 h) neuronal cell damage, either in close proximity to the brain surface (basal cortex) or in deep brain regions (hippocampus). A potential explanation could be that even SAH of minor extent can cause acute vasoconstriction, marked CPP, and subsequent rCBF decline [31].

Initial increase in ICP is considered to play an important role in the pathophysiology of EBI [5]. Prior studies have noted that a rapid increase in ICP triggers sympathetic nerve activity [34] decreases CBF, and upregulates contractile receptors in cerebral arteries [31] and therefore potentially can cause brain damage [14,34]. Despite these findings, we were not able to demonstrate a relationship between the peak ICP and the degree of neurodegeneration and number of cells with DNA damage found 24 h after SAH.

### CPP depletion as parameter for the severity of EBI after SAH

A possible explanation for the missing correlation between peak ICP and brain damage might be that peak ICP does not reflect the actual perfusion shortage during the hyperacute phase of SAH. Individual variations in baseline MABP and intensity of Cushing reflex at the time of bleeding influenced CPP depletion during SAH. CPP challenge was best represented by relative CPP reduction during the first minutes of SAH but not by peak ICP.

The amount of subarachnoid blood also seems not to be reliable for assessment of severity of EBI after SAH. Within the very first phase after the onset of experimental SAH, large blood volumes stream into the subarachnoid space [22]. After a phase of compensatory mechanisms, any further small change in intracranial volume then causes rapid increase in ICP, as well as a large CPP reduction, dependent on the degree of the Cushing reflex. This could explain why we recorded moderate and severe SAH blood scores even in animals with only minor CPP challenges. In addition, one needs to keep in mind that the degree of cisternal SAH is performed by visual inspection. It would have been favorable to either quantify the amount of SAH by determining the hemoglobin concentration in various cisterns [13] or to directly measure the blood flow in the shunt during acute SAH [22].

It also has to be acknowledged that the significant correlation between hemodynamic events and signs of neuronal damage in our study does not allow drawing causal conclusion. Our study has rather hypothesis-generating character. In this respect, we believe that also the lack of correlation between peak ICP/amount of subarachnoid blood and degree of early neuronal cell damage is an important information. It would be of much interest to verify the presented results by a modified study design in which different ICP values are generated with the same amount of blood. The blood injection technique could generate various degrees of peak ICP (by varying the injection time) with the same amount of blood; however, the ICP profiles would differ significantly among animals.

## Conclusions

The severity of EBI in terms of neuronal cell death and neurodegeneration correlates with the degree of hyperacute CPP challenge. Initial global ischemia, however, is not solely responsible for EBI. These results suggest that other processes, potentially triggered by hyperacute CPP depletion, play a major role in the onset of EBI. The total amount of subarachnoid blood and peak ICP failed as a surrogate marker for the severity of EBI.

## Additional files

Additional file 1
**This file contains supplementary figures.**
Additional file 2
**This file contains supplementary tables.**
