# Supplementary material for: Early brain injury linearly correlates with reduction in cerebral perfusion pressure during the hyperacute phase of subarachnoid hemorrhage
Source: Intensive Care Med Exp. 2014 Nov 30;2:30. doi: 10.1186/s40635-014-0030-1 (PMC4512974; doi:10.1186/s40635-014-0030-1)

**Supplemental Material**

**Supplemental Figure 1** **-** SAH blood score. The chiasmatic and pre-chiasmatic cisterns (I), basal cistern (II), prepontine and interpeduncular cisterns (III), and cistern magna (IV) underwent separate analysis of the subarachnoid blood clot according to a 3-point scoring system. ACA = anterior cerebral artery. BA = basilar artery. ICA = internal cerebral artery. MCA = middle cerebral artery. PCA = posterior cerebral artery. SCA= superior cerebellar artery.


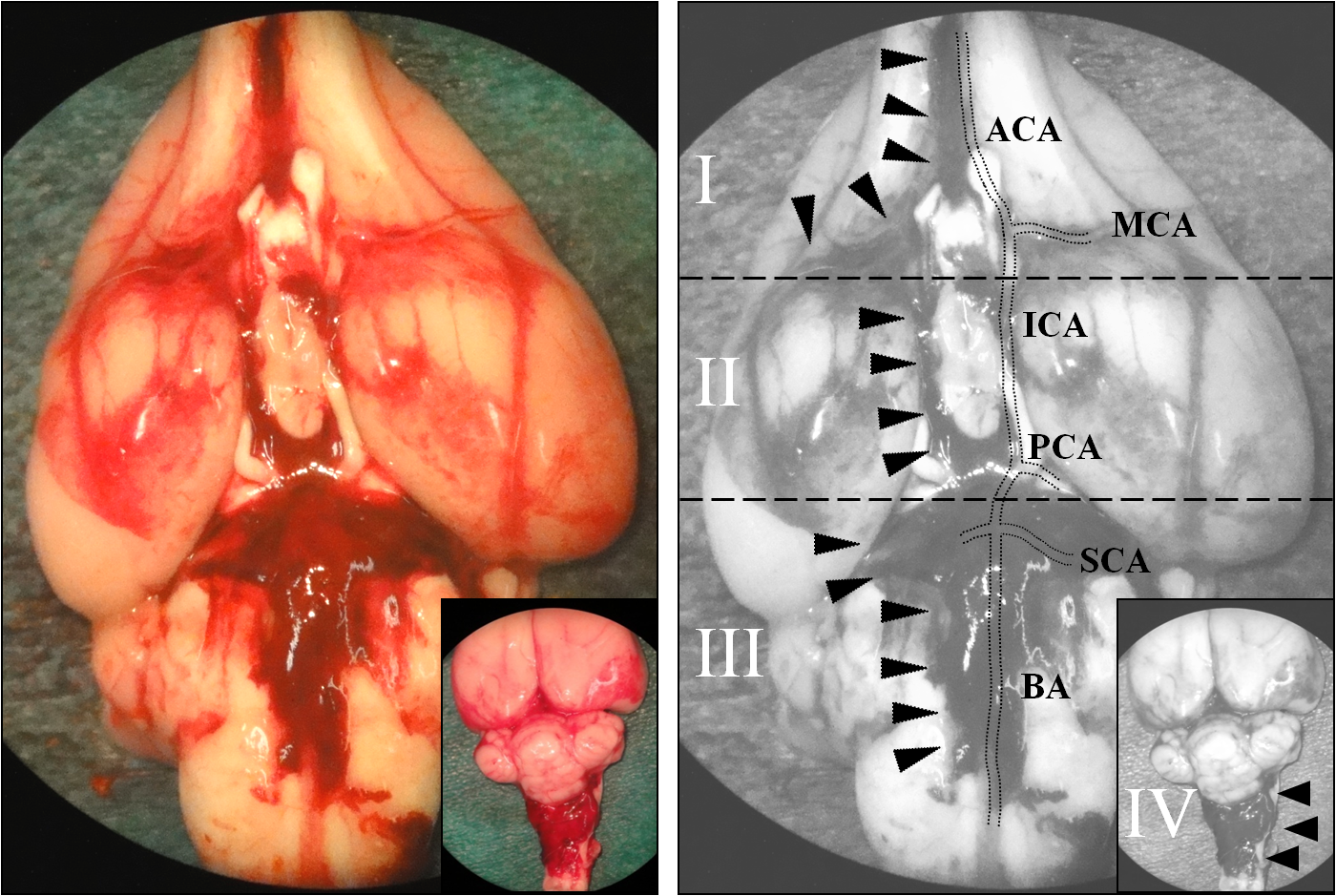


**Supplemental Figure 2 -** No linear correlation was found between the SAH blood score and the relative CPP depletion within the first 3 minutes (reg coeff r=0.31, r^2^=0.10, p>0.5; Panel A). There was also no correlation between the SAH sum core and relative CPP at the time of maximal depletion (reg coeff r=0.21, r^2^=0.43, p>0.1; Panel B).


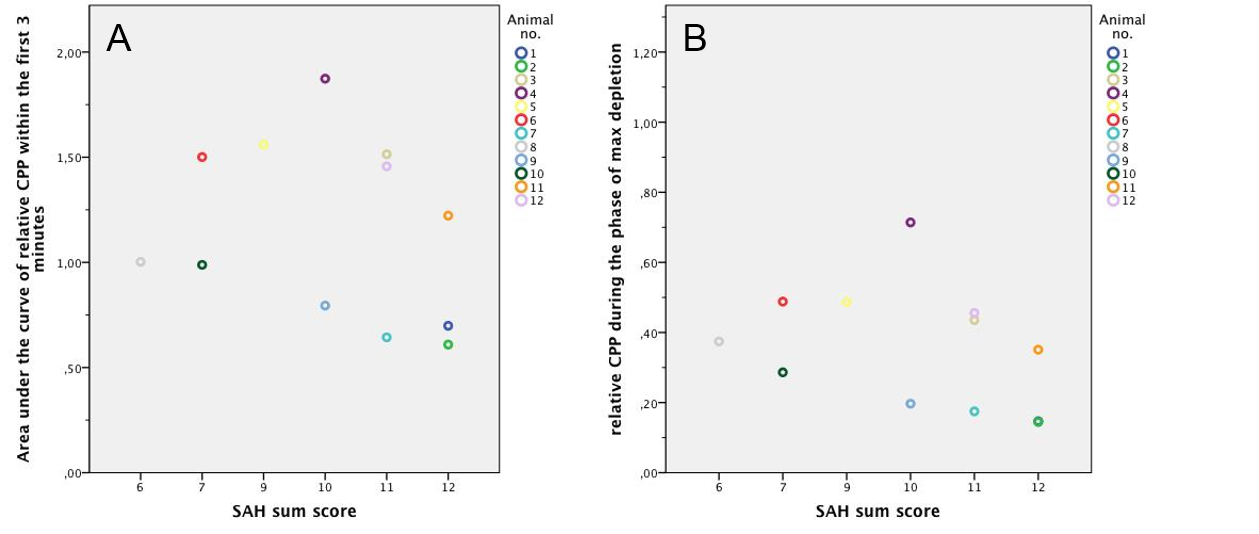


**Supplemental Figure 3 -** There was also no correlation found between the SAH blood score and mean total amount of both hemispheres of TUNEL- or FJB-positive cells. These findings were true regardless of whether the hippocampus or the basal cortex was examined. The linear regression coefficients were weak (r=0.46 (A) for FJB-positive cells in the hippocampus formation and r=0.34 (C) in the basal cortex region; r=0.01 (B) for TUNEL-positive cells in the hippocampus formation and r=0.40 (D) in the basal cortex region) and probability values were not significant (p>0.5).


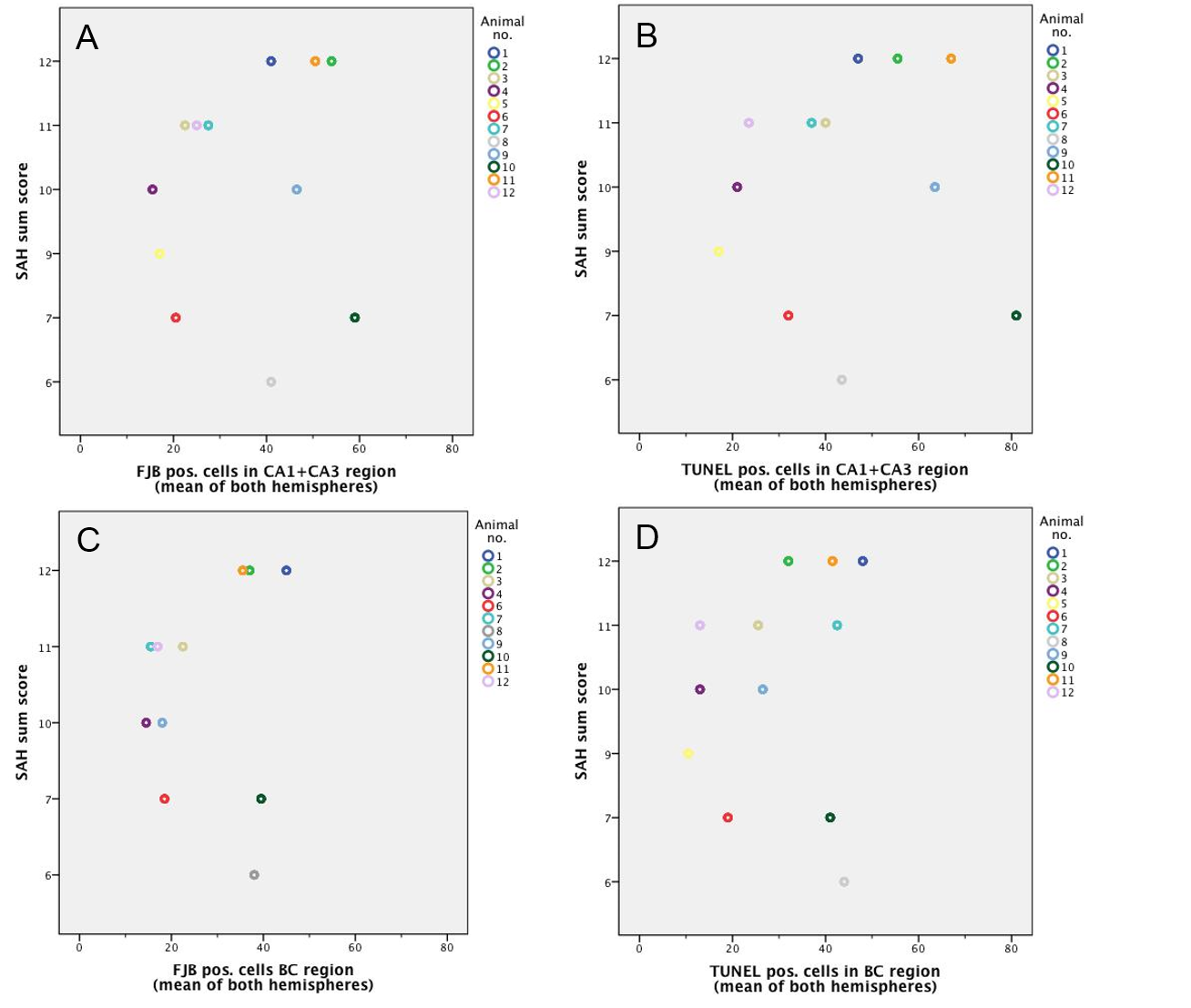

Supplement: Additional file 1 — This file contains supplementary figures. [file 40635_2014_30_MOESM1_ESM.doc]
