# Supplementary material for: Early brain injury linearly correlates with reduction in cerebral perfusion pressure during the hyperacute phase of subarachnoid hemorrhage
Source: Intensive Care Med Exp. 2014 Nov 30;2:30. doi: 10.1186/s40635-014-0030-1 (PMC4512974; doi:10.1186/s40635-014-0030-1)
Supplement: Additional file 2 — This file contains supplementary tables. [file 40635_2014_30_MOESM2_ESM.docx]

**Supplemental Table 1**

| **Relative CPP depletion** | **n** | **Mean ± SD cell count of both hemispheres** | | | |
| --- | --- | --- | --- | --- | --- |
|  |  | **Hippocampus (CA1 and CA3)** | | **Basal cortex** | |
|  |  | **TUNEL** | **FJB** | **TUNEL** | **FJB** |
| **Controls** | 5 | 33 ± 10 | 20 ± 4 | 21 ± 8 | 19 ± 3 |
| **Mild** | 5 | 27 ± 9 | 20 ± 4 | 16 ± 6 | 18 ± 3 |
| **Moderate** | 3 | 64 ± 19 | 50 ± 9 | 42 ± 2 | 38 ± 2 |
| **Severe** | 4 | 51 ± 11 | 42 ± 11 | 37 ± 10 | 29 ± 14 |

| **Dependent variable** | | **Rel. CPP depletion** | | **Mean difference** | **Standard error of the mean** | **P value** | **95% confidence interval** | |
| --- | --- | --- | --- | --- | --- | --- | --- | --- |
|  |  |  |  |  |  |  | **Lower** | **Upper** |
| **H i p p o c a m p u s** | **FJB pos. cells**  **(mean of both hemispheres)** | **0** | 1 | 0.3 | 4.55 | 1 | -13.85 | 14.45 |
|  |  |  | **2** | **-29.77** | **5.26** | **0.001** | **-46.11** | **-13.43** |
|  |  |  | **3** | **-21.85** | **4.83** | **0.003** | **-36.86** | **-6.85** |
|  |  | **1** | **2** | **-30.07** | **5.26** | **0.001** | **-46.40** | **-13.73** |
|  |  |  | **3** | **-22.15** | **4.83** | **0.003** | **-37.16** | **-7.15** |
|  |  | 2 | 3 | 7.92 | 5.50 | 1 | -9.17 | 25 |
|  | **TUNEL pos. cells**  **(mean of both hemispheres)** | **0** | 1 | 6.50 | 7.54 | 1 | -16.93 | 29.93 |
|  |  |  | **2** | **-30.63** | **8.71** | **0.023** | **-57.69** | **-3.58** |
|  |  |  | 3 | -17.55 | 7.99 | 0.282 | -42.4 | 7.3 |
|  |  | **1** | **2** | **-37.13** | **8.71** | **0.006** | **-64.19** | **-10.08** |
|  |  |  | 3 | -24.05 | 7.99 | 0.061 | -48.9 | 0.8 |
|  |  | 2 | 3 | 13.08 | 9.11 | 1 | -15.21 | 41.38 |

**Supplemental Table 2**

| **B a s a l C o r t e x** | **TUNEL pos. cells**  **(mean of both hemispheres)** | **0** | 1 | 5.10 | 4.55 | 1 | -9.03 | 19.23 |
| --- | --- | --- | --- | --- | --- | --- | --- | --- |
|  |  |  | **2** | **-20.87** | **5.25** | **0.01** | **-37.18** | **-4.55** |
|  |  |  | **3** | **-15.95** | **4.82** | **0.034** | **-30.94** | **-0.97** |
|  |  | **1** | **2** | **-25.97^*^** | **5.25** | **0.002** | **-42.28** | **-9.65** |
|  |  |  | **3** | **-21.05^*^** | **4.82** | **0.005** | **-36.04** | **-6.07** |
|  |  | 2 | 3 | 4.92 | 5.49 | 1 | -12.14 | 21.98 |
|  | **FJB pos. cells**  **(mean of both hemispheres)** | **0** | 1 | .48 | 5.13 | 1 | -15.71 | 16.66 |
|  |  |  | **2** | **-19.07** | **5.59** | **0.031** | **-36.69** | **-1.45** |
|  |  |  | 3 | -10.28 | 5.13 | 0.411 | -26.46 | 5.91 |
|  |  | **1** | **2** | **-19.54** | **5.85** | **0.035** | **-37.97** | **-1.12** |
|  |  |  | 3 | -10.75 | 5.41 | 0.422 | -27.81 | 6.31 |
|  |  | 2 | 3 | 8.79 | 5.85 | 0.95 | -9.64 | 27.22 |

Independent variables: Relative CPP depletion: 0 = control group; 1 = mild (relative CPP < 0.25); 2 = moderate (relative CPP > 0.25 <0.4); 3 = severe (relative CPP > 0.4). Significant differences are marked in bold.
